# Predictive value of the combination of SMAD4 expression and lymphocyte infiltration in malignant transformation of oral leukoplakia

**DOI:** 10.1002/cam4.1005

**Published:** 2017-03-03

**Authors:** Junki Sakata, Ryoji Yoshida, Yuichiro Matsuoka, Masashi Nagata, Akiyuki Hirosue, Kenta Kawahara, Takuya Nakamura, Masafumi Nakamoto, Masatoshi Hirayama, Nozomu Takahashi, Hikaru Nakashima, Hidetaka Arita, Hidenao Ogi, Akimitsu Hiraki, Masanori Shinohara, Hideki Nakayama

**Affiliations:** ^1^ Department of Oral and Maxillofacial SurgeryFaculty of Life SciencesKumamoto UniversityKumamotoJapan; ^2^ Section of Oral OncologyDepartment of Oral and Maxillofacial SurgeryFukuoka Dental CollegeFukuokaJapan; ^3^ Itoh Dent‐Maxillofacial HospitalKumamotoJapan

**Keywords:** Lymphocyte infiltration, malignant transformation, oral leukoplakia, oral squamous cell carcinoma, SMAD4

## Abstract

Oral leukoplakia (OL) is a common, potentially malignant disorder of the oral cavity. SMAD4 was initially identified as a tumor suppressor and central mediator of transforming growth factor (TGF)‐*β* signaling. In this study, we aimed to determine the expression patterns of SMAD4 in OL, its relationship with the degree of inflammation, and its clinical implications as a biomarker for OL malignant transformation. A total of 150 patients with OL were enrolled in this study. Paraffin‐embedded sections obtained from biopsy or resection specimens were subjected to immunohistochemical analysis. Associations among the status of epithelial SMAD4 expression, stromal lymphocyte infiltration, and malignant transformation of OL were examined. Malignant transformation was significantly associated with the status of SMAD4 expression (*P *=* *0.0017) and lymphocyte infiltration status (*P *=* *0.0054). Cox regression analysis, based on the event‐free survival (EFS), revealed that a low SMAD4 expression was a significant prognostic factor in OL patients (hazard ratio, 2.632; *P *=* *0.043). In addition, a low SMAD4 expression was closely correlated with high lymphocyte infiltration (*P *=* *0.00035), resulting in a significant correlation between the combination of low SMAD4 expression and high lymphocyte infiltration with malignant transformation of OL (*P *=* *0.00027). The combination of the status of epithelial SMAD4 expression and stromal lymphocyte infiltration may be a useful biomarker for predicting malignant transformation in OL patients. These results suggest that not only epithelial SMAD4 loss, but also stromal features, may regulate the risk of malignant transformation of OL.

## Introduction

Oral leukoplakia (OL) is a potentially malignant disorder defined as a predominantly white lesion of the oral mucosa that cannot be characterized as any other definable lesion 1, 2. Recent studies have reported a malignant transformation rate of approximately 11% in well‐defined cohorts of OL patients 3, 4. At present, the assessment of the grade of dysplasia is regarded as the gold standard for predicting the risk of the malignant transformation in OL. On the other hand, the presence of inter‐ and intraobserver variation is a major obstacle when assessing the histopathological severity of epithelial dysplasia 5. Therefore, the predictive value of dysplasia for malignant transformation in OL is not always reliable. Objective biomarkers that are independent of histopathological assessment are needed to properly assess and guide therapy in OL patients before the onset of oral squamous cell carcinoma (OSCC), a major cause of mortality worldwide.

SMAD4 was first identified as a tumor suppressor in pancreatic cancer 6, and subsequently was characterized as a key molecule in transforming growth factor (TGF)‐*β* signaling 7. SMAD4 plays a supplemental role in TGF‐*β*/bone morphogenic protein signaling by forming a trimeric complex with receptor‐activated SMADs, (i.e., SMAD2 and 3, or SMAD1 and 5) and translocating into the nucleus. In the nucleus, the SMAD trimeric complex binds to the SMAD‐binding element of target genes involved in a wide variety of cancer‐related processes 8. Numerous studies have clearly demonstrated that SMAD4 loss has both initiation and promotion effects on tumorigenesis in various tissues 9. With respect to the role of SMAD4 in head and neck squamous cell carcinoma (HNSCC), including OSCC, it has been reported that the loss of SMAD4 causes spontaneous HNSCC in a mouse model 10, and that the loss of SMAD4 proteins is also observed in clinical OSCC 10, 11. On the other hand, although SMAD4 loss is known to play an important role in the malignant progression of OSCC, the clinical significance of SMAD4 in the malignant transformation of OL has not been fully elucidated.

Chronic inflammation generates a tumor‐specific microenvironment that is favorable for cellular transformation and the progression of malignant properties of a variety of cancers, including OSCC 12, 13. Previously, Kitamura and colleagues have reported that SMAD4 loss in intestinal epithelia resulted in aggressive invasive behaviors associated with increased inflammation 14. SMAD4 loss in oral epithelia is also reported to lead to marked inflammation that is closely associated with the development of spontaneous HNSCC 10. Although these data suggest the close relationship between SMAD4 loss‐related inflammation and carcinogenesis, little is known about the association between SMAD4 expression and inflammation in precancerous lesions such as leukoplakia of the oral cavity.

In this study, we aimed to determine the expression of SMAD4 in OL lesions, its relationship with the degree of inflammation, and its clinical implications as a biomarker for OL malignant transformation.

## Material and Methods

### Patients and specimens

Histopathologically diagnosed cases of OL and OSCC were analyzed in this study. For the clinicopathological analysis, OL tissue samples were obtained from 150 patients, and primary cancerous tissue samples were obtained from 36 OSCC patients. All patients were treated at Kumamoto University Hospital from November 2000 to November 2011. Tissue samples derived from biopsy or excisional biopsy specimens were used for immunohistochemical analyses. Samples were fixed in 10% neutral‐buffered formalin, embedded, sectioned, and stained with hematoxylin and eosin (H&E) as previously described 15. A histological diagnosis of each sample was made according to World Health Organization criteria 2. Patients with lesions having more than moderate epithelial dysplasia were recommended for surgical resection. On the other hand, patients who refused surgical treatment and patients with no or mild epithelial dysplasia were followed up every 1–6 months for clinical observation. The nature and aims of the study were explained to all patients, and all gave their informed consent for this study.

### Immunohistochemical staining

SMAD4 protein expression in OL and OSCC tissue sections was validated by immunohistochemistry (IHC) as described earlier 15. Briefly, the tissue sections (5 *μ*m) were deparaffinized and subsequently rehydrated using different gradients of alcohol and probed with anti‐SMAD4 antibody (H‐552, Santa Cruz Biotechnology, Inc., CA) by incubating at 4°C overnight in a humid chamber. This was followed by sequential 60‐min incubations with secondary antibodies (EnVision + System‐HRP Labeled Polymer, Dako), and visualization with Liquid DAB + Substrate Chromogen System (Dako). All slides were lightly counterstained with hematoxylin for 30 s prior to dehydration and mounting.

### Assessment of immunohistochemical staining and stromal inflammatory response

Three independent observers, who had no knowledge of the patients’ clinical status, interpreted the immunohistochemical data in a blinded fashion. For each specimen, one score was assigned according to the percentage of positive cells: <5%: 1 point; 6–35%: 2 points; 36–70%: 3 points; and >71%: 4 points. A second score was assigned according to the intensity of the staining, with negative staining equaling 1 point, weak staining equaling 2 points, moderate staining equaling 3 points, and strong staining equaling 4 points. SMAD4 expression scores were then calculated by multiplying the two scores described above. If the expression score was ≥4, the tissue was considered as high expression. To assess the stromal inflammatory response, lymphocyte infiltration status was examined by three independent observers. The presence of a well‐defined infiltration of lymphocytes confined to the superficial part of the connective tissue was considered as high lymphocyte infiltration. On the other hand, no or slight lymphocyte infiltration was considered as low lymphocyte infiltration. When the assessment of immunohistochemical staining and/or the stromal inflammatory response was different between the examiners, a unified evaluation was performed based on a consultation between the examiners.

### Statistical analysis

The chi‐square test was used to determine the associations between malignant transformation of OL and the clinicopathological variables or SMAD4 expression status. The differences in mean values between two groups were statistically analyzed using the Mann–Whitney U test. Event‐free survival (EFS) was defined as the time from biopsy to the date of malignant transformation. The Kaplan–Meier method was used to estimate the probability of EFS as a function of time, and the log‐rank test was used to determine the correlation between the SMAD4 expression status and lymphocyte infiltration with malignant transformation. A multivariate survival analysis was performed using the Cox regression model to study the effects of malignant transformation factors on EFS. All *P*‐values were based on two‐tailed statistical analyses, and *P* < 0.05 were considered to be statistically significant (*, *P *<* *0.05 and **, *P *<* *0.01). The statistical analyses were completed using the JMP 9 software program (SAS Institute Inc., Cary, NC).

## Results

### Patient clinicopathological characteristics

Detailed clinicopathological characteristics and follow‐up information for each patient are presented in Table S1. Of the 150 patients with OL, 76 (50.7%) were male and 74 (49.3%) were female with ages ranging from 19 to 87 years old (median: 62.3 years). Among the 150 OL lesions, 64 (42.7%) were located on the gingiva, 52 (34.7%) were located on the tongue, 21 (14.0%) were located on the buccal mucosa, and 13 (8.7%) were located on other oral cavity sites. During the follow‐up period (average follow‐up, 34.0 months), 23 of 150 (15.3%) OL lesions demonstrated malignant transformation after the initial diagnosis. The 150 patients with OL in the present cohort were grouped as malignant transformed (*n* = 23) and untransformed (*n* = 127) cases. The detailed clinicopathological characteristics of the patients with OSCC are summarized in Table S2.

### Immunohistochemical staining patterns of SMAD4

**Figure 1:**
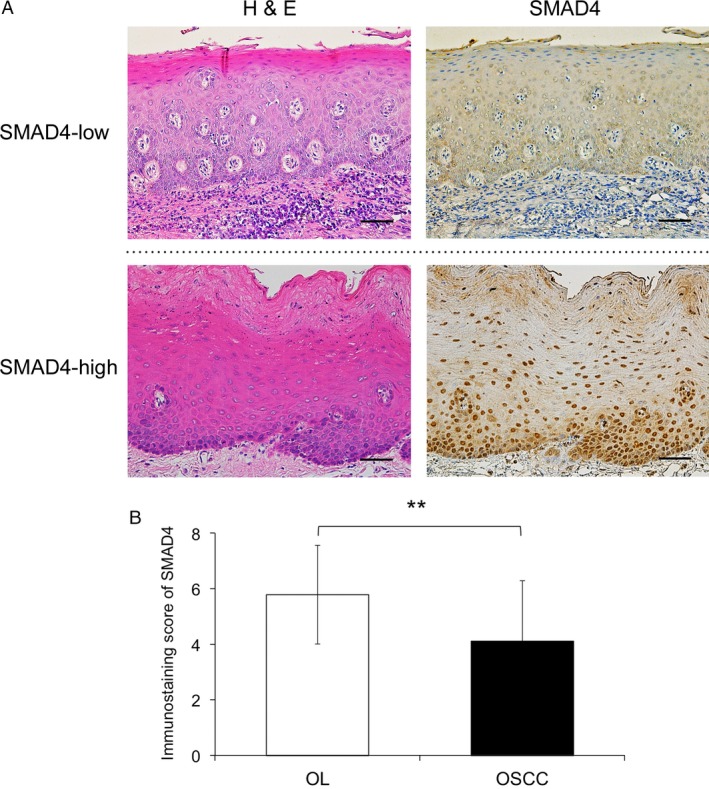
Immunohistochemical staining of SMAD4 in oral leukoplakia and oral squamous cell carcinoma tissues. (A) Representative immunohistochemical staining patterns of SMAD4 are shown according to the expression status. The sections were stained with hematoxylin and eosin (H&E) and anti‐SMAD4 polyclonal antibodies. In the oral leukoplakia (OL) specimens, SMAD4 was mainly expressed in the nucleus of the epithelial cells. Original magnification, ×200, scale bar = 100 *μ*m. (B) Immunostaining scores obtained from each pathological group were calculated and statistically analyzed by the Mann–Whitney U test. The *y* axis shows the mean values of the SMAD4 immunostaining scores. **, *P *<* *0.01 oral squamous cell carcinoma, OSCC.

Because previous studies clearly reported that SMAD4 loss or reduction is found in squamous cell carcinomas (SCCs) in different tissues including OSCC 16, 17, we focused on SMAD4 expression in precancerous lesions. We examined the expression levels of SMAD4 protein in the biopsy or resection specimens obtained from the 150 OL patients using immunohistochemical staining. Representative immunohistochemical staining results for SMAD4 are shown in Figure 1. SMAD4 immunoreactivity was observed mainly in the nucleus of epithelial cells, and the expression status of SMAD4 was diverse. Therefore, we classified the patients into two groups based on the immunostaining score for SMAD4 expression as follows: SMAD4‐low expression group (score <4) or SMAD4‐high expression group (score ≥4). Of the 150 OL cases, 84 cases (56.0%) showed SMAD4‐high expression and 66 cases (44.0%) showed SMAD4‐low expression. In addition, there was a significant difference in SMAD4 immunostaining scores between OL tissues and OSCC tissues (Fig. 1B).

### Reduced SMAD4 expression and high lymphocyte infiltration are associated with malignant transformation of OL

In order to determine the malignant transformation‐associated factors including SMAD4 in OL, we examined the relationships between clinicopathological variables and malignant transformation (Table 1). Of the 150 OL cases, 23 cases (15.3%) developed malignant transformation of OL. Malignant transformation was associated with the status of both SMAD4 expression (*P *=* *0.0017) and lymphocyte infiltration (*P *=* *0.0054). On the other hand, there were no significant associations of malignant transformation with age, sex, oral subsite, and dysplasia grade. Although we had only limited data on smoking and alcohol intake, malignant formation was not associated with these variables (Table S3).

**Table 1:** Correlation between malignant transformation and clinicopathological factors in 150 oral leukoplakia patients

Characteristics	Total	Malignant transformation		*P*‐value
		Yes *n* (%)	No *n* (%)	
	150	23 (15.3)	127 (84.7)	
Age (years)				
Median	62.3	66	61.7	
Range	19–87	47–87	19–87	
≤65	85	12 (14.1)	73 (85.9)	0.637
>65	65	11 (16.9)	54 (83.1)	
Sex				
Male	76	9 (11.8)	67 (88.2)	0.229
Female	74	14 (18.9)	60 (81.1)	
Oral subsite				
Gingiva	64	11 (17.2)	53 (82.8)	0.128
Tongue	52	11 (21.2)	41 (78.8)	
Buccal mucosa	21	1 (4.8)	20 (95.2)	
Others	13	0 (0)	13 (100)	
Grade of dysplasia				
None	101	13 (12.9)	88 (87.1)	0.427
Mild	37	7 (18.9)	30 (81.1)	
Moderate, severe	12	3 (25.0)	9 (75.0)	
SMAD4 expression				
High	84	6 (7.1)	78 (92.9)	0.0017^a^
Low	66	17 (25.8)	49 (74.2)	
Lymphocyte infiltration				
Low	51	2 (3.9)	49 (96.1)	0.0054^a^
High	99	21 (21.2)	78 (78.8)	

The chi‐square test was used to examine the correlation between malignant transformation and clinicopathological factors in 150 oral leukoplakia patients.

a *P *<* *0.01.

### SMAD4 expression status affects the malignant transformation rate of OL

**Figure 2:**
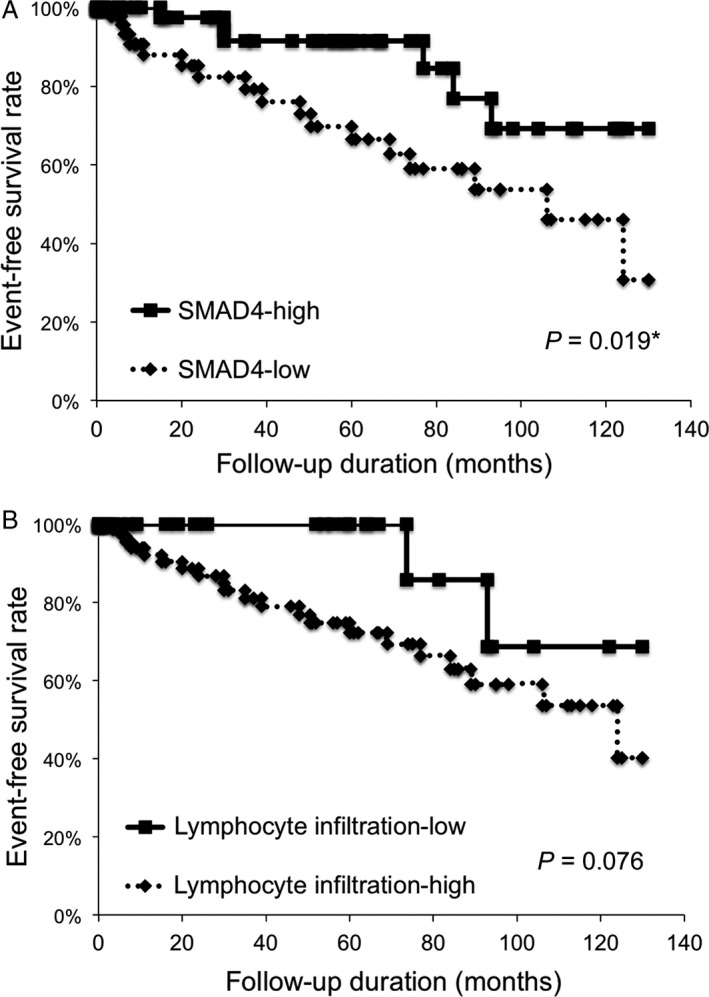
SMAD4 expression status affects the malignant transformation rate of oral leukoplakia. In the Kaplan–Meier survival analysis of patients with oral leukoplakia **(** OL), patients were divided into two groups based on the SMAD4 immunostaining scores (‐low or ‐high expression groups) or the lymphocyte infiltration status (lymphocyte infiltration‐low or ‐high groups). (A) Event‐free survival (EFS) of the 150 OL patients based on the status of SMAD4 expression. *, *P *<* *0.05. (B) EFS of the 150 OL patients based on the status of lymphocyte infiltration. NS, no significance.

In order to determine whether SMAD4 or lymphocyte infiltration affects the EFS rate of OL, we assessed the relationships between the clinicopathological factors and the time of malignant transformation using the Kaplan–Meier method. In the 150 patients with OL, the median EFS time from diagnosis was 34.0 months (95% CI = 27.5–40.4). The EFS in the SMAD4‐low group was significantly lower than that seen in the SMAD4‐high group (*P *=* *0.019; Fig. 2A). On the other hand, the EFS rate in the lymphocyte infiltration‐high group tended to be lower than that in the lymphocyte infiltration‐low group, but this difference was not statistically significant (*P *=* *0.076, Fig. 2B).

### Reduction of SMAD4 expression is a possible predictive factor of OL malignant transformation

In order to determine independent malignant transformation factors for EFS, multivariate analysis was performed using a Cox proportional hazards regression model. Multivariate analysis revealed that SMAD4 expression status was a significant predictive factor for the malignant transformation of OL (hazard ratio, 2.632; *P *=* *0.043) (Table 2). On the other hand, age, sex, oral subsite, dysplasia grade, and lymphocyte infiltration were not significant factors for EFS in OL patients (Table 2).

**Table 2:** The results of the multivariate analysis of malignant transformation factors by the Cox proportional hazards regression model

Characteristics	Assigned score	Event‐free survival	*P*‐value
		Hazard ratio (95% CI)	
Age (years)			
≤65	0	1.902 (0.794–4.902)	0.151
>65	1		
Sex			
Male	0	1.098 (0.469–2.705)	0.831
Female	1		
Oral subsite			
Tongue	0	1.305 (0.546–3.151)	0.547
Others	1		
Grade of dysplasia			
None	0	1.191 (0.492–2.792)	0.689
Mild, moderate, severe	1		
SMAD4 expression			
High	0	2.632 (1.031–7.654)	0.043^a^
Low	1		
Lymphocyte infiltration			
Low	0	2.670 (0.729–17.24)	0.152
High	1		

CI, confidence interval.

a *P *<* *0.05.

### Reduction of SMAD4 expression is correlated with inflammatory stromal features

**Figure 3:**
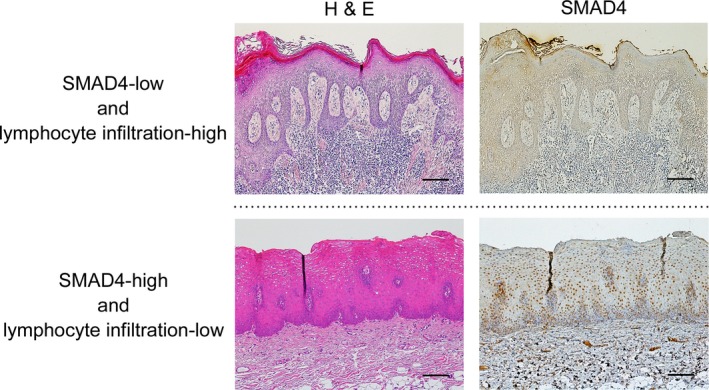
Reduction of SMAD4 expression is correlated with inflammatory stromal features. Representative immunohistochemical staining patterns of SMAD4 and the status of lymphocyte infiltration are shown according to the expression status. The sections were stained with hematoxylin and eosin (H&E) and anti‐SMAD4 polyclonal antibodies. Original magnification, ×100, scale bar = 50 μm.

In order to elucidate the clinical significance of SMAD4 expression status in OL patients, we examined the correlation between SMAD4 expression and clinicopathological variables (Table 3). The expression status of SMAD4 was closely correlated with the status of lymphocyte infiltration (*P *=* *0.00029). The representative images of the status of SMAD4 expression and lymphocyte infiltration are shown in Figure 3. On the other hand, there was no correlation between SMAD4 expression status and age, sex, oral subsite, or dysplasia grade.

**Table 3:** Correlation between SMAD4 expression and clinicopathological factors in 150 oral leukoplakia patients

Characteristics	Total	SMAD4 expression		*P*‐value
		High *n* (%)	Low *n* (%)	
	150	84 (56.0)	66 (44.0)	
Age (years)				
Median	62.3	66	61.7	
Range	19–87	47–87	19–87	
≤65	85	49 (57.6)	36 (42.4)	0.642
>65	65	35 (53.8)	30 (46.2)	
Sex				
Male	76	42 (11.7)	34 (88.3)	0.854
Female	74	42 (19.2)	32 (80.8)	
Oral subsite				
Gingiva	64	33 (51.6)	31 (48.4)	0.586
Tongue	52	25 (48.1)	27 (51.9)	
Buccal mucosa	21	14 (66.7)	7 (33.3)	
Others	13	12 (92.3)	1 (0.08)	
Grade of dysplasia				
None	101	55 (54.5)	46 (45.5)	0.859
Mild	37	22 (59.5)	15 (40.5)	
Moderate, severe	12	7 (58.3)	5 (41.7)	
Lymphocyte infiltration				
Low	51	39 (76.5)	12 (23.5)	0.00029**
High	99	45 (45.5)	54 (54.5)	

The chi‐square test was used to examine the correlation between SMAD4 expression and clinicopathological factors in 150 OL patients.

a *P *<* *0.01.

### Combination of SMAD4 expression and lymphocyte infiltration status is a statistically significant predictive marker for malignant transformation of OL

**Figure 4:**
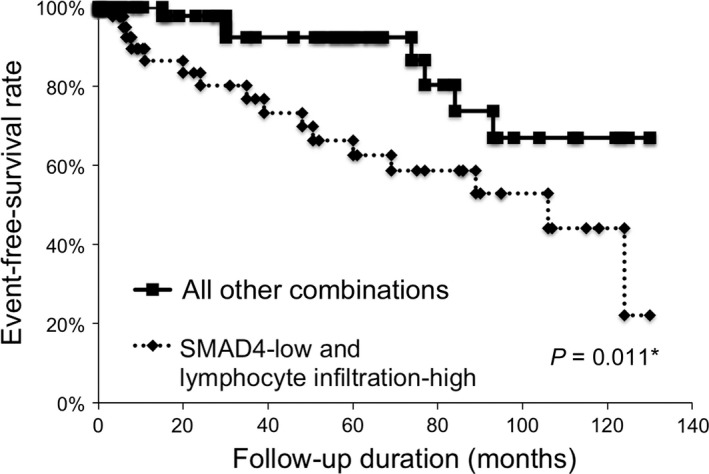
Combination of SMAD4 expression and lymphocyte infiltration status is useful predictive marker for the malignant transformation of oral leukoplakia. In the Kaplan–Meier survival analysis of patients with oral leukoplakia (OL), patients were divided into two groups. One group was the patients with SMAD4‐low expression and lymphocyte infiltration‐high, and the other group was all other combinations. The graph shows the event‐free survival (EFS) of the 150 patients with OL based on the status of SMAD4 expression and lymphocyte infiltration. *, *P *<* *0.05.

In order to assess the relationships among SMAD4 expression, lymphocyte infiltration, and malignant transformation, we investigated the predictive value of the combination of SMAD4 expression and lymphocyte infiltration status. As a result, malignant transformation was found to be significantly associated with the combination of low SMAD4 expression and high lymphocyte infiltration (*P *=* *0.00027, Table 4). In addition, the EFS rate of patients with both low SMAD4 expression and high lymphocyte infiltration was significantly lower than that of the remainder of the cohort (*P *=* *0.011, Fig. 4). Based on these results, the combination of SMAD4 expression status and lymphocyte infiltration had a statistically significant predictive value for the malignant transformation of OL.

**Table 4:** Correlation between malignant transformation and the combination of SMAD4 expression and lymphocyte infiltration in 150 oral leukoplakia patients

Characteristics	Total	Malignant transformation		*P*‐value
		Yes *n* (%)	No *n* (%)	
	150	23 (15.3)	127 (84.7)	
SMAD4‐low and lymphocyte infiltration‐high	54	16 (29.6)	38 (70.3)	0.00027^a^
Others	96	7 (7.3)	89 (92.7)	

The chi‐square test was used to examine the correlation between malignant transformation and the combination of SMAD4 expression and lymphocyte infiltration in 150 OL patients.

a *P *<* *0.01.

## Discussion

To date, several factors including age, sex, smoking, oral subsite, and dysplasia grade have been suggested as predictors of the risk of malignant transformation of OL 18, 19. However, the predictive value of these risk factors is not established. Therefore, the identification of useful biomarkers independent of clinical characteristics is needed to improve our assessment of the risk of malignant transformation. Although data regarding smoking and alcohol intake were insufficient in our cohort (Table S1), we found a significant relationship between low SMAD4 expression and the malignant transformation of OL. Previously, Xia and colleagues 20 have reported that high SMAD4 expression was a useful predictor of the malignant transformation of OL. However, in that study, various grades of dysplasia were observed in all OL tissues, in contrast to this study in which 67.3% of OL lesions showed no dysplasia, possibly accounting for this discrepancy.

Numerous studies have clearly demonstrated that SMAD4 loss has both initiating and promoting effects on tumorigenesis in many tissues 9. Our data are in line with these previous observations (Fig. 1B). In SMAD4‐deficient tissues, decreased expression of TGF‐β target molecules regulating growth inhibition is reported to be an important mechanism underlying SMAD4 loss‐associated tumorigenesis 21. On the other hand, Bornstein et al. postulated that other mechanisms are active in carcinogenesis of the oral epithelia by studying inducible head and neck cancer‐specific SMAD4 knockout mice (HN‐Smad4^−/−^) 10. In that model, both normal and precancerous tissues from HN‐Smad4^−/−^ mice exhibited increased genomic instability, which was linked to a disruption of the Fanconi anemia/BRCA DNA repair pathway. In addition to the increasing genomic instability in oral epithelial cells, they also proposed that marked inflammation in the stroma adjacent to both normal buccal mucosa and precancerous lesions also plays an important role in the development of HNSCC. Taken together, our data raise the possibility that the loss of SMAD4 in the oral epithelia of OL lesions evokes chronic inflammation in the stroma, which promotes malignant transformation leading to the development of OSCC.

The close association between inflammation and the risk of cancer has been clarified in recent decades 12. With respect to OSCC development, chronic exposure to carcinogens such as tobacco, alcohol, oncogenic viruses, and inflammation can increase the risk of oral cancer 22, 23. Indeed, chronic inflammatory conditions, such as oral lichen planus and oral ulcers, related to repetitive tissue injury have been proposed to be related to the pathogenesis of OSCC 23. On the other hand, although chronic inflammatory conditions of the oral mucosa are common, it is evident that inflammatory processes alone rarely induce the development of cancer 24. Recently, much attention has been paid to the reciprocal interactions between epithelial cells and their stromal cells in the microenvironment of oral disease, including OSCC 25, 26, 27. Therefore, it is conceivable that an alteration of epithelial cells, such as SMAD4 loss, may be an important event leading to the malignant transformation of OL.

To the best of our knowledge, we demonstrate for the first time that decreased SMAD4 expression can be used as a biomarker to predict the risk of malignant transformation in patients with OL. Importantly, the prediction is made independent of the dysplasia grade, which is regarded as a potential clinical biomarker. Furthermore, we showed that the combination of a low SMAD4 expression and high lymphocyte infiltration is a better predictor for the malignant transformation of OL than either factor alone.

A major limitation of this study is that we focused on the significance of only SMAD4 among the many molecules involved in TGF‐*β* signaling. However, SMAD4 expression is known to be associated with other activated SMAD proteins linked to the Fanconi anemia/BRCA DNA repair pathway. Further investigations are needed to elucidate the detailed mechanisms underlying SMAD4 loss‐related malignant transformation of OL. In addition, aberrations of other DNA repair‐related molecules that might be correlated with OL malignant transformation need to be assessed in future studies. Furthermore, another limitation of this study is the limited information regarding clinical characteristics, including lesion size and the use of tobacco and alcohol by the patients. Therefore, a study of OL malignant transformation with a comparative analysis of SMAD4 status and clinical data is necessary.

## Conflict of Interest

No potential conflicts of interest were disclosed.

## Supporting information


**Table S1.** Characteristics in 150 oral leukoplakia patients.

**Table S2.** Characteristics in 36 oral squamous cell carcinoma patients.

**Table S3.** Correlation between malignant transformation and clinicopathological factors in 150 oral leukoplakia patients.

**Table S4.** Correlation between SMAD4 expression and clinicopathological factors in 150 oral leukoplakia patients.
