# Supplementary material for: Predictive value of the combination of SMAD4 expression and lymphocyte infiltration in malignant transformation of oral leukoplakia
Source: Cancer Med. 2017 Mar 3;6(4):730–8. doi: 10.1002/cam4.1005 (PMC5387127; doi:10.1002/cam4.1005)
Supplement: Supplementary file 1 — Table S1. Characteristics in 150 oral leukoplakia patients. [file CAM4-6-730-s001.docx]

| Table S1. Characteristics in 150 oral leukoplakia patients | |
| --- | --- |
| Characteristics | n (%) |
| Age (years) |  |
| Median | 62.3 |
| Range | 19 - 87 |
| ≤ 65 | 85 (56.7) |
| > 65 | 65 (43.3) |
| Sex |  |
| Male | 76 (50.7) |
| Female | 74 (49.3) |
| Oral subsite |  |
| Gingiva | 64 (42.7) |
| Tongue | 52 (34.7) |
| Buccal mucosa | 21 (14.0) |
| Others | 13 (8.7) |
| Grade of dysplasia |  |
| None | 101 (67.3) |
| Mild | 37 (24.7) |
| Moderate, severe | 12 (8.0) |
| SMAD4 expression |  |
| High | 84 (56.0) |
| Low | 66 (44.0) |
| Lymphocyte infiltration |  |
| Negative | 51 (34.0) |
| Positive | 99 (66.0) |
| Smoking |  |
| Never | 16 (10.7) |
| Past and present | 15 (10.0) |
| Data missing | 119 (79.3) |
| Alcohol intake |  |
| Never | 13 (8.7) |
| Past and present | 14 (9.3) |
| Data missing | 123 (82.0) |
| Malignant transformation |  |
| Yes | 23 (15.3) |
| No | 127 (84.7) |
