# Supplementary material for: Predictive value of the combination of SMAD4 expression and lymphocyte infiltration in malignant transformation of oral leukoplakia
Source: Cancer Med. 2017 Mar 3;6(4):730–8. doi: 10.1002/cam4.1005 (PMC5387127; doi:10.1002/cam4.1005)
Supplement: Supplementary file 2 — Table S2. Characteristics in 36 oral squamous cell carcinoma patients. [file CAM4-6-730-s002.docx]

| Table S2. Characteristics in 36 oral squamous cell carcinoma patients | |
| --- | --- |
| Characteristics | n (%) |
| Age (years) |  |
| Median | 68.4 |
| Range | 36 - 86 |
| ≤ 65 | 12 (33.3) |
| > 65 | 24 (66.7) |
| Sex |  |
| Male | 18 (50.0) |
| Female | 18 (50.0) |
| Primary site |  |
| Tongue | 5 (13.9) |
| Mandible | 11 (30.6) |
| Maxilla | 5 (13.9) |
| Buccal mucosa | 8 (22.2) |
| Oral floor | 5 (13.9) |
| Palate | 2 (5.6) |
| pT-stage |  |
| T1, T2 | 17 (47.2) |
| T3 | 11 (30.6) |
| T4 | 8 (22.2) |
| pN-stage |  |
| N0 | 19 (52.8) |
| ≥ N1 | 17 (47.2) |
| Pathological stage |  |
| Ⅱ | 11 (30.6) |
| Ⅲ | 9 (25.0) |
| Ⅳ | 16 (44.4) |
| Differentiation |  |
| Well | 29 (80.6) |
| Moderate | 5 (13.9) |
| Poor | 2 (5.6) |
