# Supplementary material for: Predictive value of the combination of SMAD4 expression and lymphocyte infiltration in malignant transformation of oral leukoplakia
Source: Cancer Med. 2017 Mar 3;6(4):730–8. doi: 10.1002/cam4.1005 (PMC5387127; doi:10.1002/cam4.1005)
Supplement: Supplementary file 3 — Table S3. Correlation between malignant transformation and clinicopathological factors in 150 oral leukoplakia patients. [file CAM4-6-730-s003.docx]

| Table S3. Correlation between malignant transformation and clinicopathological factors in 150 oral leukoplakia patients | | | | |
| --- | --- | --- | --- | --- |
|  |  | Malignant transformation | |  |
| Characteristics | Total | Yes n (%) | No n (%) | *P* - value |
|  | 150 | 23 (15.3) | 127 (84.7) |  |
| Smoking | | | | |
| Never | 16 | 11 (68.8) | 5 (31.2) | 0.108 |
| Past and present | 15 | 6 (40.0) | 9 (60.0) |  |
| Data missing | 119 |  |  |  |
| Alcohol intake | | | | |
| Never | 13 | 8 (61.5) | 5 (38.5) | 0.816 |
| Past and present | 14 | 8 (57.1) | 6 (42.9) |  |
| Data missing | 123 |  |  |  |
| The chi-square test was used to examine the correlation between malignant transformation and clinicopathologic factors in 150 OL patients. | | | | |
