# Supplementary material for: Predictive value of the combination of SMAD4 expression and lymphocyte infiltration in malignant transformation of oral leukoplakia
Source: Cancer Med. 2017 Mar 3;6(4):730–8. doi: 10.1002/cam4.1005 (PMC5387127; doi:10.1002/cam4.1005)
Supplement: Supplementary file 4 — Table S4. Correlation between SMAD4 expression and clinicopathological factors in 150 oral leukoplakia patients. [file CAM4-6-730-s004.docx]

| Table S4. Correlation between SMAD4 expression and clinicopathological factors in 150 oral leukoplakia patients | | | | |
| --- | --- | --- | --- | --- |
|  |  | SMAD4 expression | |  |
| Characteristics | Total | High n (%) | Low n (%) | *P* - value |
|  | 150 | 84 (56.0) | 66 (44.0) |  |
| Smoking | | | | |
| Never | 16 | 5 (31.3) | 11 (68.7) | 0.213 |
| Past and present | 15 | 8 (53.3) | 7 (46.7) |  |
| Data missing | 119 |  |  |  |
| Alcohol intake | | | | |
| Never | 13 | 4 (30.8) | 9 (69.2) | 0.310 |
| Past and present | 14 | 7 (50.0) | 7 (50.0) |  |
| Data missing | 123 |  |  |  |
| The chi-square test was used to examine the correlation between Smad4 expression and clinicopathological factors in 150 OL patients. | | | | |
